# Graphene oxide from silk cocoon: a novel magnetic fluorophore for multi-photon imaging

**DOI:** 10.1007/s13205-013-0128-2

**Published:** 2013-03-24

**Authors:** Manas Roy, Tejas Sanjeev Kusurkar, Sandeep Kumar Maurya, Sunil Kumar Meena, Sushil Kumar Singh, Niroj Sethy, Kalpana Bhargava, Raj Kishore Sharma, Debabrata Goswami, Sabyasachi Sarkar, Mainak Das

**Affiliations:** 1 Department of Chemistry, Indian Institute of Technology Kanpur, Kanpur, 208016 Uttar Pradesh India; 2 Department of Biological Sciences and Bioengineering, Bioelectricity, Green Energy, Physiology and Sensor Group, Indian Institute of Technology Kanpur, Kanpur, 208016 Uttar Pradesh India; 3 Center for Laser Technology, Indian Institute of Technology Kanpur, Kanpur, 208016 Uttar Pradesh India; 4 Department of Electrical Engineering, Indian Institute of Technology Kanpur, Kanpur, 208016 Uttar Pradesh India; 5 MEMS and Photonics Division, Solid State Physics Laboratory, Defense Research Development Organization, Timarpur, Delhi, 110054 India; 6 Peptide and Proteomics Division, Defense Institute of Physiology and Allied Sciences, Defense Research Development Organization, Timarpur, Delhi, 110054 India; 7 Department of Chemistry, Division of Physical Chemistry, Electrochemical Materials Research Group, University of Delhi, Delhi, 110007 India; 8 Department of Chemistry, Bengal Engineering and Science University, Shibpur, Botanical Garden, Howrah, 711103 West Bengal India; 9 Design Program, Indian Institute of Technology Kanpur, Kanpur, 208016 Uttar Pradesh India

**Keywords:** Bio-imaging, Fluorophore, Graphene oxide, Magnetism, Multi-photon imaging, Silk cocoon

## Abstract

In this work, we synthesized graphene oxide from silk cocoon embarking its new dimension as a magnetic fluorophore when compared with its present technical status, which at best is for extracting silk as a biomaterial for tissue engineering applications. We produced graphene oxide by pyrolysing the silk cocoon in an inert atmosphere. The collected raw carbon is oxidized by nitric acid that readily produces multilayer graphene oxide with nano carbon particulates. Structural properties of the graphene oxide were analyzed using scanning electron microscopy, transmission electron microscopy, Fourier transform infra-red spectroscopy, and Raman spectroscopy. The oxidized sample shows remarkable fluorescence, multi-photon imaging and magnetic properties. On increasing the excitation wavelength, the fluorescence emission intensity of the graphene oxide also increases and found maximum emission at 380 nm excitation wavelength. On studying the two photon absorption (TPA) property of aqueous graphene oxide using Z-scan technique, we found significant TPA activity at near infrared wavelength. In addition, the graphene oxide shows ferromagnetic behavior at room temperature. The observed fluorescence and magnetic property were attributed to the defects caused in the graphene oxide structure by introducing oxygen containing hydrophilic groups during the oxidation process. Previously silk cocoon has been used extensively in deriving silk-based tissue engineering materials and as gas filter. Here we show a novel application of silk cocoon by synthesizing graphene oxide based magnetic-fluorophore for bio-imaging applications.

**Electronic supplementary material:**

The online version of this article (doi:10.1007/s13205-013-0128-2) contains supplementary material, which is available to authorized users.

## Introduction

Carbon, the sixth most abundant element, exhibits the unique property of forming a wide variety of structures and allotropes. Because pre-historic times, only two allotropes of carbon are known widely: graphite and diamond. However, provoked by the discovery of zero-dimensional allotrope (0D) fullerenes by Kroto et al. (1985), there is immense interest for nano-carbon materials in recent years. In 1991, Iijima (1991) discovered one-dimensional allotrope (1D) of graphite consisting of closed tubular structures known as carbon nano tubes, which was followed by the discovery of two dimensional (2D) allotrope of carbon known as grapheme (Novoselov et al. 2004). This remarkable breakthrough shattered the prior belief that 2D-thin atomic layer cannot exist under normal condition.

Graphene is a flat monolayer sheet of the graphite. It is a 2D hexagonal lattice consisting of a network of *sp*^2^ bonded carbon atoms. It is the “thinnest material” that is stable in free form (Novoselov et al. 2004). Because its discovery, graphene has received enormous research interest due to its unusual band structure, unique electronic properties (Schwierz 2010), excellent thermal conductivity (Balandin et al. 2008) and outstanding mechanical properties (Lee et al. 2008). Graphene was first successfully synthesized in 1859 through repeated oxidation of graphite in 1:3 mixtures of fuming nitric acid (HNO_3_) and potassium chlorate (KClO_3_) resulting in brilliantly transparent yellow pallets (Brodie 1859). In 1961, thin black graphite lamella (thickness 3.7 Å) was synthesized by oxidation of graphite with potassium permanganate (KMnO_4_) and potassium chlorate (KClO_3_) by dissolving them in concentrated sulfuric acid (H_2_SO_4_) (Bohem et al. 1962). In the past few years, various techniques, such as mechanical exfoliation techniques, chemical vapour deposition (CVD) technique and other chemical techniques were developed for synthesis of multilayer graphene to single layer graphene. Most of the techniques developed in past years have utilized very high sophistication and cost. However recently Ruan et al. (2011) reported a low cost synthesis method of high quality graphene monolayer from food sources, insects and waste. Till date, graphene oxide synthesis methods have been time consuming and are not completely environmental friendly (Guo and Dong 2011). Herein, we describe a novel and efficient approach of low-cost synthesis of graphene by pyrolysis of Tasar silk cocoon, followed by its oxidation using nitric oxide to produce graphene oxide. Silk derived from cocoon has traditionally been used in cosmetic and personal applications. It has found limited technical applications in the area of tissue engineering, optics (Tsioris et al. 2010), biomedical devices and as a gas filter (Roy et al. 2012). Our work brings in the novel dimension of the potential use of silk cocoon as magnetic–fluorophore biomaterial.

Furthermore, over the past few years, magnetic property of carbon-based nano materials has received a lot of attention. Fullerene-based organic materials show ferromagnetism at low temperature (Allemand et al. 1991). Even at room temperature, magnetic signals have been observed in rhombohedral C60 (Makarova et al. 2001). It is well established that pure carbon materials are diamagnetic in nature (Ramirez et al. 1994). Electronic instabilities due to bonding defects may be responsible for magnetic interactions in different forms of carbon nano-materials (Makarova 2004). The presence of unpaired electrons in a graphitic network can also give rise to ferromagnetism. Theoretically, it was proved that the introduction of *sp*^2^/*sp*^3^ mixed phase may be responsible for ferromagnetic signal of nano-carbon material (Kim et al. 2003). Recently, our group observed both room temperature and low temperature ferromagnetic behavior of water-soluble carbon nano tubes (Dubey et al. 2005). Wang et al. (2008) found room temperature ferromagnetic behavior of grapheme.

Graphene oxide has also find applications in the field of cellular and molecular biology. Using graphene’s fluorescence quenching property, scientists have developed novel technologies to detect low concentration of biological macromolecules, such as DNA and protein (Lu et al. 2009). Owing to its biocompatibility and less toxicity, graphene oxide has been used for imaging mammalian cells (Qian et al. 2012). Animal cells are also imaged with ‘graphene quantum dots’ (Zhu et al. 2011). Graphene has shown promise in the field of electrochemistry, hydrogen storage, supercapacitor and solar cell technology (Guo and Dong 2011). In most of the above studies, graphene has been derived from chemical and physical processing of graphite.

Using natural resource, here in, we have synthesized and characterized our synthesized graphene oxides structurally by scanning electron microscopy (SEM), transmission electron microscopy (TEM), and Fourier transform infrared spectroscopy (FTIR) and Raman spectroscopy. The synthesized graphene oxide also exhibit excellent fluorescence property and two-photon activity at near-IR (NIR) excitations (800 nm), thus making it a very strong candidate for two-photon microscopy applications. Magnetic property measurements of graphene oxide showed ferromagnetic behavior at room temperature indicating its excellent potential in magneto-optic studies also.

## Experimental

### Materials

All chemical reagents were of analytical grade, and used without further purification and Tasar silk cocoon were collected from the state of Chhattisgarh in India as previously described (Roy et al. 2012).

### Synthesis of graphene and graphene oxide from Tasar silk cocoon

The collected cocoons were grounded in mortar and pestle. Three grams of grounded Tasar silk cocoon was pyrolysed in a muffle furnace at 400 °C for 2 h in argon (Ar) atmosphere. This resulted in the formation of black solid which was collected at room temperature. The powdered raw carbon was taken in a thimble made of Whatman filter paper. Then, this raw carbonized cocoon was subjected to soxhlet purification. Continuous washing of raw carbon powder were done by petroleum ether followed by acetone. The raw carbonized cocoon was oxidized by treating with concentrated nitric acid (HNO_3_) for 24 h. Then, the acid was decanted off and the brown mass was thoroughly washed with distilled water till it was free from acid and becomes neutral. The brown residue was finally dried in vacuum and subjected for further analysis.

### Scanning electron microscopy

Morphology of both the raw carbonized cocoon (containing graphene) and oxidized carbonized cocoon (containing graphene oxide) were characterized using SUPRA 40VP field emission scanning electron microscope (FESEM) equipped with energy dispersive X-ray (EDX) facility (Carl Zeiss NTS GmbH). To analyze the soxhlet purified raw carbonized cocoon (containing graphene), 3 mg of sample was taken in 4 ml isopropanol solution and sonicated for 30 min. 50 μl of the sonicated solution was placed on brass stubs and vacuum dried. For analyzing graphene oxide, 3 mg sample was taken in 4 ml isopropanol and sonicated for 3 min. 50 μl of the sonicated solution was placed on brass stubs and vacuum dried and subjected to SEM analysis.

### Transmission electron microscopy (TEM)

TEM was performed using Tecnai 20 G2 300 kV, STWIN model. An acceleration voltage of 200 kV was used for TEM analysis. Samples for TEM analysis were prepared by dropping 5 μl of the re-dispersed raw and oxidized carbon on to a carbon-coated copper TEM grid with a mess size of 400.

### FT-IR

FT-IR spectra were recorded using Bruker Vertex 70 FT-IR spectrophotometer using KBr disc.

### Raman spectroscopy

After Raman spectra were recorded using a Raman spectrometer (WITEC model) with 514 nm excitation.

### Fluorescence microscopy

Fluorescence microscopy was performed using Leica Microsystems DM 2500 microscope.

### Fluorescence emission and excitation spectroscopy

Fluorescence spectra of graphene oxide were obtained using Perkin Elmer LS 55 fluorescence spectrometer with peltier temperature programmer (PTP-1). The fluorescence excitation spectrum was recorded at the highest emission wave length of 460 nm.

### Nonlinear optical (NLO) properties

Open aperture Z-scan technique (Xu et al. 2008) was used to measure the NLO property of the prepared graphene oxide composite. Mode-locked femtosecond titanium:sapphire laser (Mira 900 oscillator) pumped with coherent Verdi frequency doubled Nd:vanadate laser was used for the Z-scan measurement using a chopper for minimal thermal effects (Choi et al. 2010). Sample solution was prepared by sonicating 5 mg of graphene oxide in 10 ml water for 40 min followed by keeping it still for ~2 h to allow the suspension to settle down and the clear solution present at the top was used in our experiments. Laser pulses at 780 nm with 4.4 nJ pulse^−1^ energy and 150 fs pulse width was focused on to the sample with a 20 cm focal lens. Across the focal plane of this lens, a 1-mm path length cuvette containing the clear aqueous sample solution was scanned in the laser propagation direction [so is the name Z scan (Xu et al. 2008)] where the Rayleigh range in our set up was 3.4 mm and the beam waist of 30 μm at 780 nm and such experimental conditions ensured that the Rayleigh range condition of Z scan (Xu et al. 2008) was satisfied. Rhodamine-6G was used as a reference for calibrating the experimental results. An open aperture Z-scan trace for the sample is shown in the result, which when fitted to the theoretical model (Xu et al. 2008) for two-photon absorption (TPA) gives a measure of the nonlinearity in terms of the TPA coefficient.

### Magnetic measurements

Magnetic measurement was performed at room temperature of 25 °C using ADE, EV7 VSM magnetometer.

## Results and discussion

### Synthesis of graphene oxide from a biological source

**Fig. 1:**
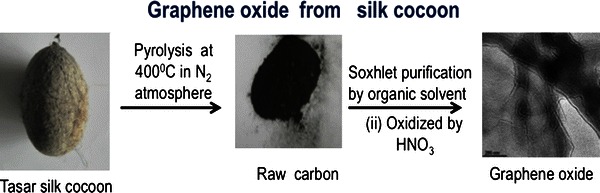
Schematic representation of synthesis of graphene oxide

Schematic representation of synthesis of graphene oxide from Tasar silk cocoon is shown in Fig. 1. The collected Tasar silk cocoons were carbonized in argon atmosphere. The obtained carbonized product termed as “raw carbon” was washed with acetone and ethanol to remove the soluble organic materials, such as poly-aromatic hydrocarbons. After oxidation with nitric acid, these raw carbon products were oxidized that results in the formation of a very thin graphene oxide layered nanostructure. Mostly, silk cocoon is used for extracting silk for tissue engineering applications, optics and biomedical devices and as gas filter. Here, we showed that silk cocoon could be used for synthesizing graphene oxide nanostructure which exhibit fluorescent and magnetic properties.

### Scanning electron microscopy

Figure 1 (Supplementary information) shows the SEM images of purified raw carbon and oxidized carbon. The raw carbon contains layers of sheets with carbon particulates and amorphous carbon. These amorphous carbons were removed during oxidation process and layered graphene oxide is separated out.

### Transmission electron microscopy

**Fig. 2:**
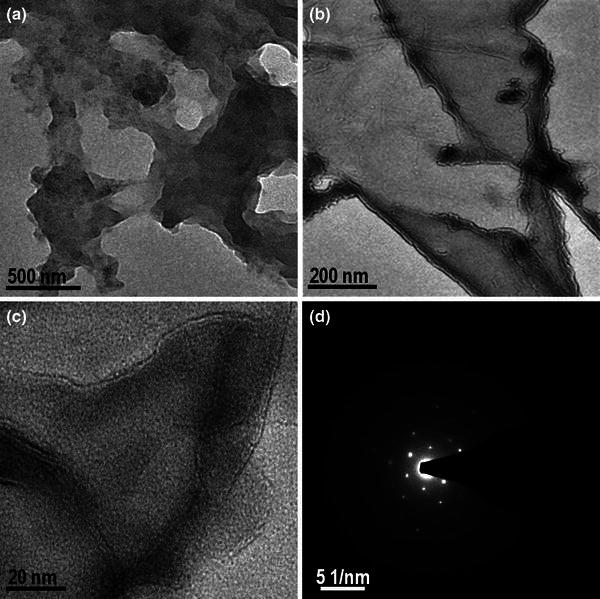
TEM images of graphene oxide **a** at 500 nm *scale bar*, **b** at 200 nm *scale bar*, **c** at 20 nm *scale bar* and **d** selected area diffraction pattern (SADP) of graphene oxide

TEM image of oxidized carbon shown in Fig. 2a–c indicates the presence of multi-layer of graphene oxide. The sheets of graphene oxide are overlapped with each other which can be observed on the contrast basis. The selected area diffraction pattern (SADP) shows presence of hexagonal lattice (Sun et al. 2010) (Fig. 2d) with typical sixfold symmetry for graphene oxide. Thus TEM investigation confirms presence of graphene oxide in the oxidized carbon.

### FT-IR spectroscopy

**Fig. 3:**
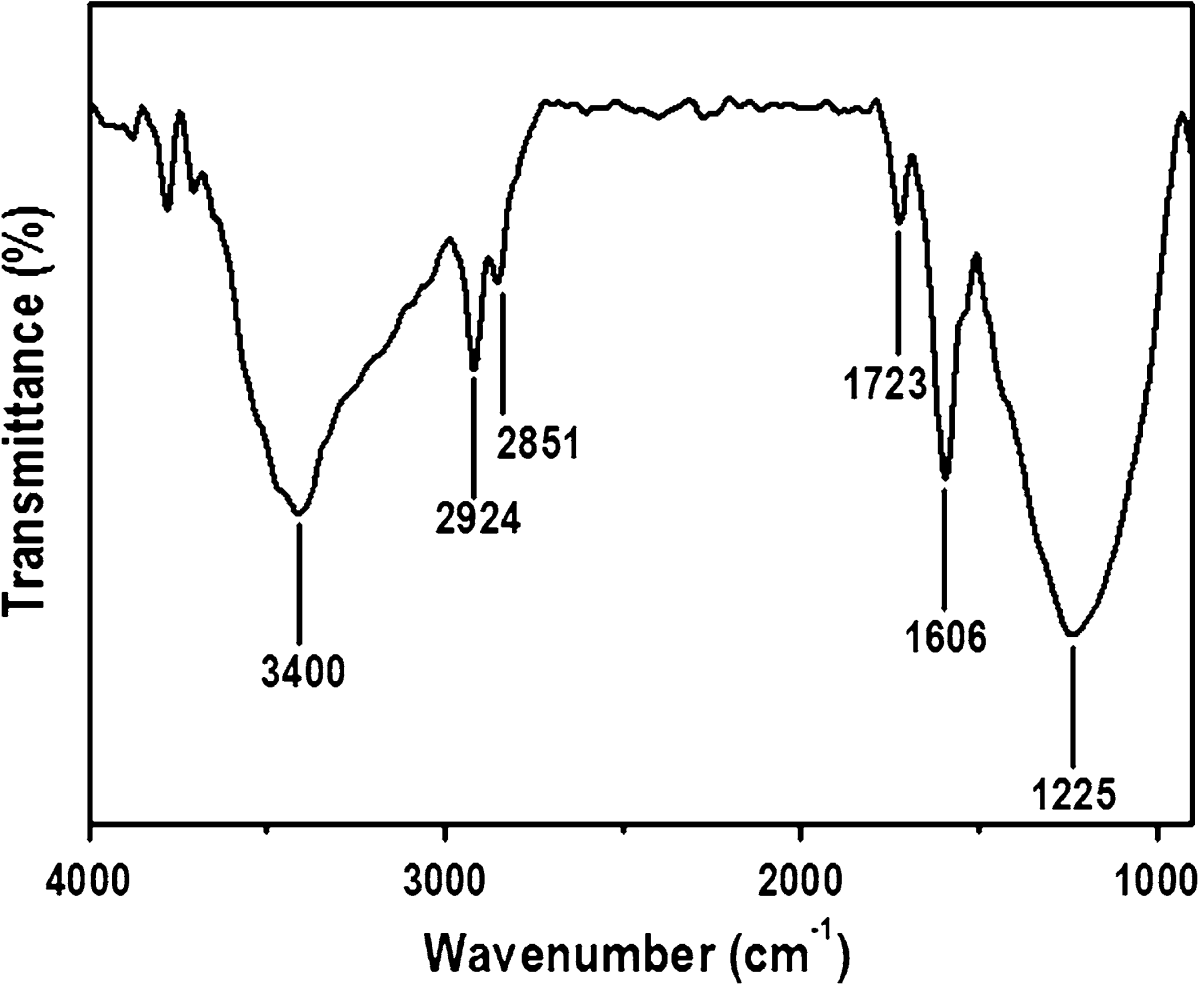
FT-IR spectra of graphene oxide

FT-IR spectroscopy was performed to study the presence of different functional groups. FT-IR spectra of graphene oxide in Fig. 3, indicates the presence of the different type of oxygen functionalities in graphene oxide (refer to supplementary information for further discussion of FT-IR data and the relevant references).

### Raman spectroscopy

**Fig. 4:**
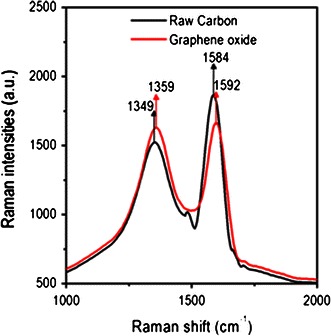
Raman spectra of raw carbonized cocoon and oxidized carbonized cocoon

To verify the structure of graphene oxide as well as to ensure the complete synthesis of graphene oxide, Raman spectroscopy has been performed, which showed the presence of *sp*^2^ and *sp*^3^ hybridized forms of carbon in the graphene oxide (Fig. 4). For comparison, Raman spectra of raw carbon is shown in Fig. 4. The Raman spectrum of soxhlet purified raw soot shows two intense peaks, centered at 1,347 (D-band) and 1,584 cm^−1^ (G-band). The D-band occurs due to the number of defects and broken symmetry of basal plane of graphitized carbon atom. The G-band corresponds to the Eg vibrational mode of *sp*^2^ hybridized carbon atoms in both rings and chain structures. The Raman spectrum of the oxidized soot is shown in Fig. 4. It has two prominent peaks at 1,592 and 1,359 cm^−1^. The first one is associated with the stretching vibrations of *sp*^2^ hybridized carbon atoms and the second one is attributed to the disorder-induced stretching modes of graphite. The D-line of oxidized soot has almost the same intensity as that of the G-line when compared with the insoluble raw soot residue (Fig. 4). It was also observed that both the D-band and G-band are slightly shifted towards higher wave numbers in graphene oxide compared to the raw soot. The blue shift of these peaks can be attributed to several possible explanations. The appearance of this band in graphite can be attributed to the presence of Raman inactive nonzero phonon density of states which lie above the G band. It becomes active due to the phonon confinement caused by the defects (Nemanich and Solin 1979; Nag et al. 2009; Eda et al. 2011). Secondly, G-band shifted to higher wave number from graphite crystal to graphene sheet. This shift may partially be responsible for the blue shift of the G band frequencies in graphene oxide, if considerable amount of unmodified graphitic areas remain (Ferrari et al. 2006). The resonance of isolated double bonds at higher frequencies than those of the G band of graphite is one of the plausible reasons. Chemical doping is also another factor for considerable shifts of band to higher wavenumbers (Pan et al. 2010).

### Fluorescence studies

**Fig. 5:**
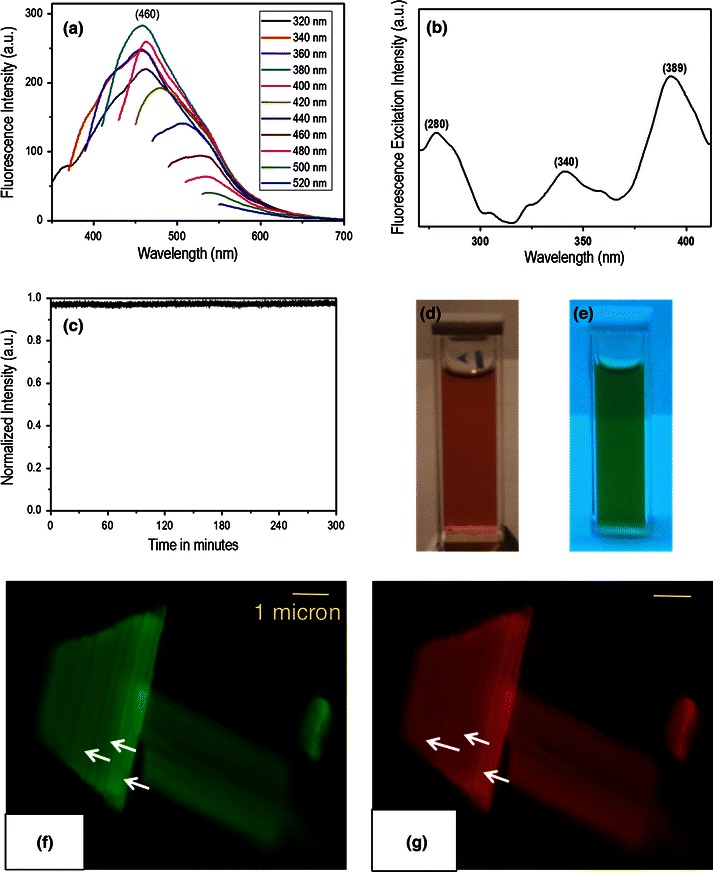
Fluorescence studies. **a** Fluorescence emission spectra of water-soluble graphene oxide under progressive 20 nm increment excitation wavelengths from 320 to 520 nm, **b** fluorescence excitation spectrum at 460 nm wavelength, **c** photo-bleaching study at 380 nm excitation wavelength, *x* axis represents time in minute, **d** graphene oxide solution under normal light, **e** and under UV light exposure, **f** and **g** fluorescence microscopic images of graphene oxide at excitation wavelength (*λ*) 488 and 532 nm respectively

The fluorescent emission spectrum of graphene oxide is shown in Fig. 5a. We tried to separate the graphene oxide layer from the carbon nano-particles by centrifugation and sonication. However, we were unable to separate them to a significant level. Hence the fluorescence properties were studied for a mixture of graphene oxide containing carbon nano-particles. We took 2 mg of oxidized carbon soot in 6 ml of water. Oxidized carbon soot shows fluorescence emission peak at 460 nm when it is excited at 320 nm. On increasing the excitation wavelength, the fluorescence emission intensity also increases and we found the maxima at 380 nm excitation wavelength. On increasing the excitation wavelength beyond 380 nm, the fluorescent emission intensity decreases though the shift in the emission wavelength continues towards higher wavelengths. The origin of fluorescence emission is yet to be explored. Literature survey points to several factors that may be responsible for the origin of fluorescence emission. The presence of carbene-like triplet ground state (*σ*^1^*π*^1^) at the zigzag sites was one of the possible explanations for the fluorescent emission (Ferrari et al. 2006; Pan et al. 2010). Another possible reason for fluorescent emission of graphene oxide could be radiative recombination of electron hole pairs, which originate within localized states. Graphene oxide contains a mixture of *sp*^2^ and *sp*^3^ bonding while graphene consist of *sp*^2^ hybridized carbon atoms. The presence of isolated finite-sized molecular *sp*^2^ cluster within the carbon–oxygen *sp*^3^ matrix in graphene oxide can lead to confinement of *π* electron within localized states and facilitating radiative recombination of electron hole pair (e–h). Radiative recombination of this e–h pair in these localized *sp*^2^ clusters give rise to fluorescence property (Loh et al. 2010). In graphene oxide, surface passivation occurs due to the formation of extensive hydrogen bonding by introduction of oxygen-containing functional group during oxidation process (Ghosh et al. 2011). The surface passivation occurring due to the introduction of such a type of covalent function group leads to the fluorescent emission of graphene oxide. Theoretically, it has been proved that materials containing smaller *sp*^2^ cluster in graphene oxide will fluoresce in the UV–visible region, whereas the larger size *sp*^2^ cluster fluoresces in the NIR region (Eda et al. 2010). The fluorescent excitation spectrum of oxidized carbon was recorded at the 460 nm emission wavelength. From Fig. 5b, it could be clearly seen that there are three peaks, respectively at, 280 (4.42 eV), 340 (3.64 eV) and 389 nm (3.18 eV). The relative differences in energy (δ*E*) of fluorescent excitation study of graphene oxide are ~0.78 eV (4.42–3.64 eV), ~1.24 eV (4.42–3.18 eV), and ~0.45 eV (3.64–3.19 eV). Hoffman demonstrated that for a triplet ground state carbene, the difference in energy (δ*E*) between *σ* and *π* orbital must be less than 1.5 eV (Graf et al. 2007). Our study shows that all the difference in energy falls in the boundary of energy difference for triplet ground state of carbene. Thus, the presence of three peaks in fluorescence excitation is not clear to us. Perhaps, the presence of different type of functional group such as carboxylic group and hydroxyl group in the surroundings environment of triplet state of carbene is responsible for such observation.

### Photobleaching study and the stability of the graphene oxide

The fluorescence intensity of graphene oxide remains almost the same on irradiation with laser of 380 nm up to 5 h as shown in Fig. 5c. Because there is no characteristic change in fluorescence intensity even after a long period of time, this indicates the stability of graphene oxide under photo bleaching conditions.

### Graphene oxide solution in normal light and in UV light

Figure 5d, e showed the photographs of graphene oxide solution in water, under normal light and under UV light exposure, respectively. Following UV exposure, we get green photo-luminescence from the solution of the synthesized graphene oxide.

### Optical fluorescence microscopy of graphene oxide sheets

Optical fluorescence microscopic images of graphene oxide particles are shown in Fig. 5f, g. The fluorescent images obtained using band pass filters of 488 and 530 nm wavelengths, respectively. In both these conditions the graphene oxide particles exhibit significant fluorescent properties.

### Nonlinear optical properties examined with two photon fluoresceence and pulse laser studies

**Fig. 6:**
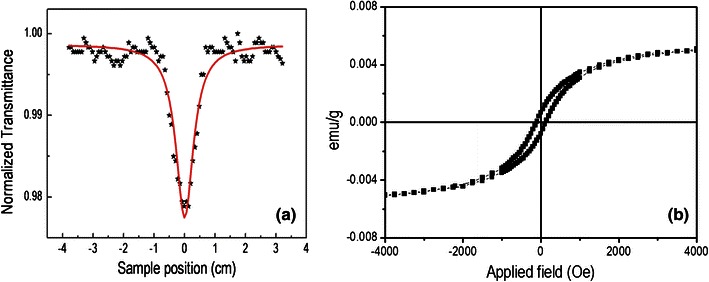
**a** Raw data (*black asterisk*) from open aperture z-scan of synthesized graphene oxide as a function of sample position (*z*). An *overlaid line plot* shows the best theoretical fit of the raw data (see text for details). **b** M–H loop measured at room temperature for the synthesized graphene oxide

The efficiency of any nonlinear two-photon material is measured in terms of the TPA coefficient (*β*) of the system. The schematic of the experimental set-up for the measurement of the optical properties is shown in Fig. 2 of supplementary material. Figure 6a shows an open aperture Z-scan trace for graphene oxide in water collected in our femtosecond experimental set-up. We fit the data to the theoretical model for TPA, which for our clear transparent sample with negligible linear absorption coefficient reduces to the following expression (Nag and Goswami 2009): 1where *T*(*z*) is the normalized energy transmittance as a function of *z* position for a sample thickness *L*; *I*_0_ is the incident laser intensity (1.09 nJ pulse^−1^) at the focus of the lens; and for the laser at wavelength *λ* (780 nm) with a minimum beam radius *ω*_0_ (30 μm) at the focus. Because all the parameters in Eq. (1) are known except *β*, fitting the experimental *T*(*z*) data at different *z* positions easily gives the value of *β* as 0.55 × 10^−9^ cm W^−1^.

### Magnetism

Room temperature ferromagnetic behavior of graphene oxide is shown in Fig. 6b. The observed coercive field (*H*_c_) of graphene oxide is 143.75 and saturation magnetization (*M*_s_) is 0.005 emu g^−1^. Low coercivity values indicating the graphene oxide is a soft magnetic material. To understand the origin ferromagnetic behavior of graphene oxide several theoretical works has been done, but it is not yet fully understood. Theoretical study has been suggested that graphene oxide show ferromagnetism due to the presence of various defects and long-range ordered coupling among these defects. In graphene oxide, the presence of C–OH, –COOH, and C=O groups which creates various defects may be responsible for ferromagnetic behavior of graphene oxide. Introduction of this hydrophilic group on the topological structures of raw carbon obtained from pyrolysis of silk cocoon may leads to increase defects due to intra or inter molecular hydrogen bonding among them.

## Conclusion

In summary, we have demonstrated the synthesis of graphene oxide from Tasar silk cocoon by pyrolyzing at 400 °C in argon atmosphere followed by its oxidation with nitric acid. Different characterization techniques were utilized to characterize the raw carbon and oxidized carbon. Synthesized water soluble graphene oxide shows remarkable fluorescence property. On increasing the excitation wave length to 380 nm, emission peak was observed at 460 nm. Its tunable fluorescence property with different wavelength can be used in applications like bio-imaging. Soft ferromagnetic behavior of graphene oxide which also exhibits single and two-photon fluorescence characteristics can open the new door for wide potential application, such as, magneto-optics, carbon-based magneto resistance, spintronics, magnetic memory devices, and so on.

## Electronic supplementary material

Below is the link to the electronic supplementary material.

Supplementary material 1 (DOC 1402 kb)
