# Supplementary material for: Graphene oxide from silk cocoon: a novel magnetic fluorophore for multi-photon imaging
Source: 3 Biotech. 2013 Mar 24;4(1):67–75. doi: 10.1007/s13205-013-0128-2 (PMC3909567; doi:10.1007/s13205-013-0128-2)
Supplement: Supplementary file 1 — Supplementary material 1 (DOC 1402 kb) [file 13205_2013_128_MOESM1_ESM.doc]

**Supplementary information**

Figure 1. SEM images of graphene oxide. (a) SEM images of raw carbonized cocoon and white arrow indicates presence of few layer graphene sheets with amorphous carbon and oxidized raw carbonized cocoon (b) sheet of graphene oxide after nitric acid treatment (scale bar 2 µm) (c) multilayer graphene oxide sheets with a width of 2.6 nm (scale bar 100 nm) (d) graphene oxide sheets with carbon particulates (scale bar 100 nm).


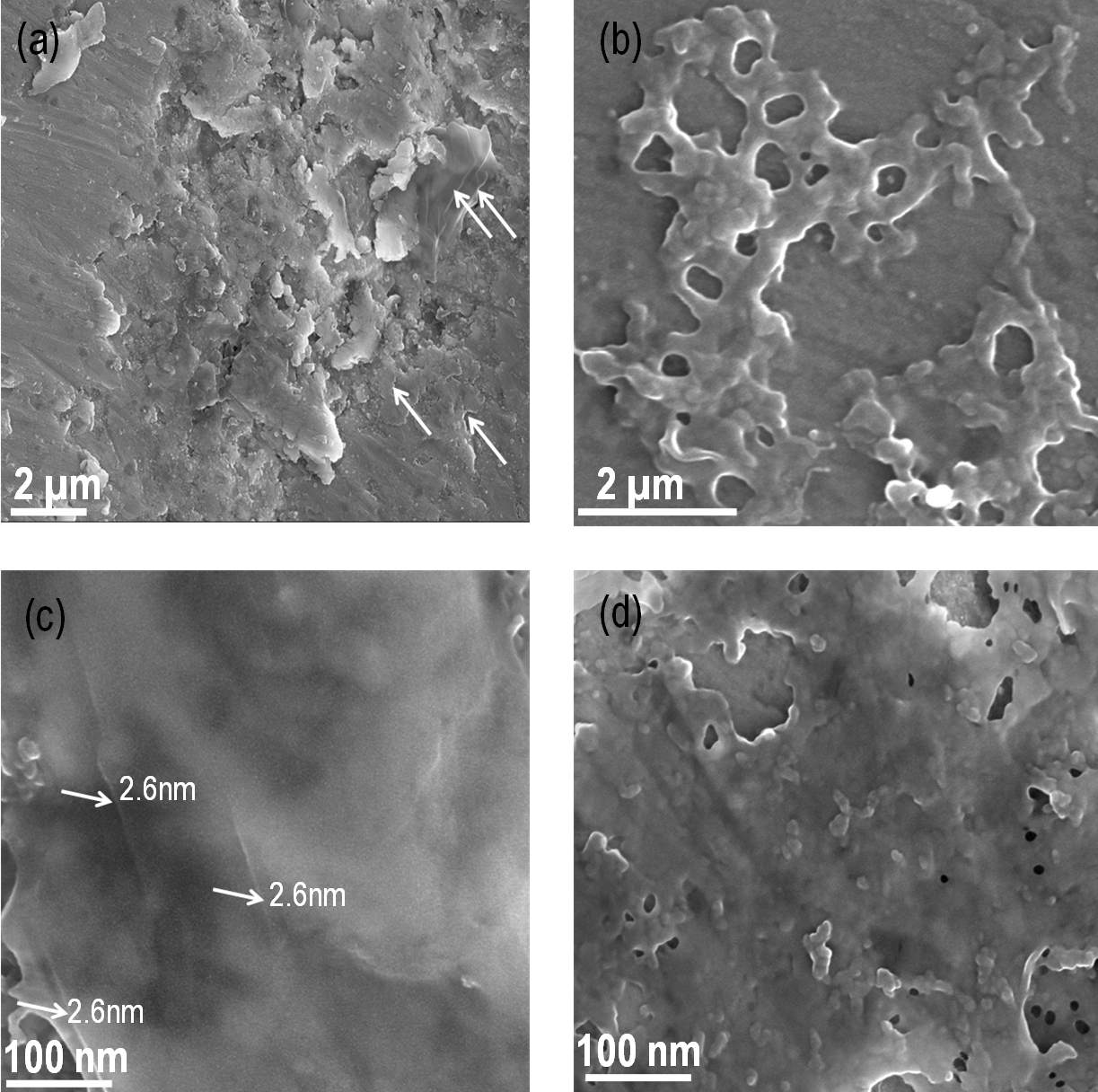
.

Figure 2. Experimental z scan setup with Femtosecond high repetition rate laser using optical chopper for the minimization of thermal effect.


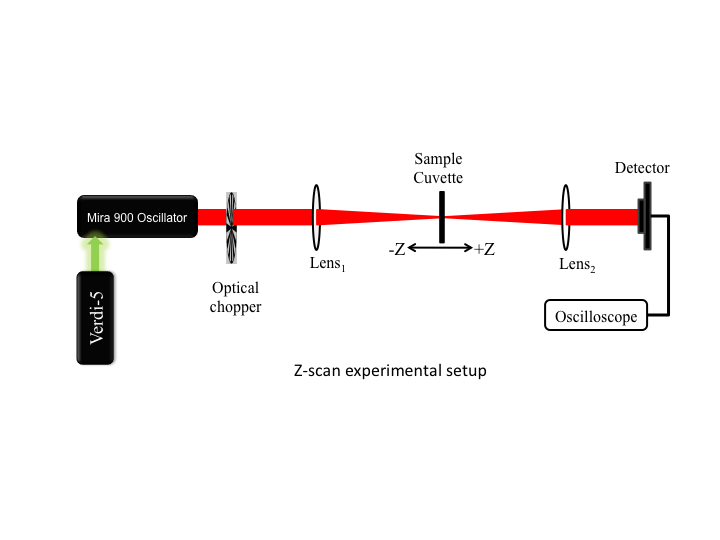


- Analysis of FT-IT data

A broad band centered at 3400 cm^-1^ is attributed due to O-H stretching vibrations. A peak centered at 1720 cm^-1^ is due to stretching vibrations from C=O bond, indicating the presence of carboxylic acid groups. Band at 1608 cm^-1^ is due to sp^2^ hybridized carbon atom. A band at 1217 cm^-1^ is associated with C-O bond stretching vibrations (Tuinstra and Koenig 1970; Kudin et al. 2008).

Relevant references:

Kudin KN, Ozbas B, Schniepp HC, Prud'Homme RK, Aksay IA, Car R (2008) Raman spectra of graphite oxide and functionalized graphene sheets. Nano Lett 8:36-41

Tuinstra F, Koenig JL (1970) Raman spectrum of graphite. J Chem Phys 53:1126
